# Older adults’ perspectives towards optimizing lifestyle behaviors and strategies to support healthy brain ageing during COVID-19 restrictions

**DOI:** 10.3389/fpubh.2023.1205001

**Published:** 2023-08-30

**Authors:** Joyce Siette, Laura Dodds, Cristy Brooks, Kay Deckers

**Affiliations:** ^1^The MARCS Institute for Brain, Behaviour and Development, Western Sydney University, Sydney, NSW, Australia; ^2^Australian Institute of Health Innovation, Macquarie University, Sydney, NSW, Australia; ^3^Translational Health Research Institute, Western Sydney University, Campbelltown, NSW, Australia; ^4^Alzheimer Centrum Limburg, Department of Psychiatry and Neuropsychology, School for Mental Health and Neuroscience (MHeNs), Maastricht University, Maastricht, Netherlands

**Keywords:** COVID-19 impact, dementia, risk reduction, lifestyle behaviors, older adults, brain health

## Abstract

**Introduction:**

It is unknown how the impact of COVID-19 restrictions has affected brain healthy behaviors that contribute to dementia risk reduction in older adults. Our aim was to explore perspectives of older adults on lifestyle behaviors that support positive brain health and dementia risk reduction during and following COVID-19 restrictions.

**Methods:**

Community-dwelling older Australians (*N* = 159) during June to October 2021 (the second wave of COVID-19 restrictions) who had taken part in a pre-post dementia risk reduction intervention program were invited to discuss the impact of COVID-19 on their lifestyle behaviors. Semi-structured interviews explored individual’s adaptability to pandemic restrictions, intended behavior changes following restrictions easing, and feedback on the effectiveness of ongoing intervention programs for sustaining brain health. Thematic data analysis was performed using a deductive approach.

**Results:**

Participants had an average age of 73.1 years (SD = 5.6; range: 65–90), majority were female (74.7%), lived in a major city (82.2%) and mean 9.5 years (SD = 1.7) of education. Older adults’ views about lifestyle prevention strategies during the pandemic were both positive (e.g., more spare time and adaptive leisure activities) and negative (e.g., social isolation, lack of motivation, adverse emotions). Participants highlighted a continuous conscious effort to adapt certain brain healthy behaviors despite the persistence of adverse impacts of COVID-19 restrictions. Participants also expressed the intention and desire to revert to their previous lifestyle before the COVID-19 pandemic or a sense of the ‘new normal’.

**Conclusion:**

This formative research will inform future interventions targeting dementia risk reduction to consider the immediate and lasting effects of COVID-19 restrictions on older adult’s lifestyle behavior.

## 1. Introduction

Globally, there is increasing evidence that modifying risk factors can potentially reduce the risk of developing dementia and support brain health in later life (1–6), with increasing awareness that dementia prevention should be targeted systematically (6–9). Several lifestyle interventions targeting multiple dementia risk factors have thus been developed and implemented (10–13). Such interventions typically focus on encouraging lifestyle changes. For example, two large scale interventions (i.e., FINGER trial (5) and Maintain Your Brain (14)) provided conservative evidence of success in improving cognitive functioning and decreasing modifiable dementia risk (2, 15, 16); and an intervention for self-management led to improvements in cardiovascular risk profiles (e.g., HATICE trial (4)). Other interventions have had no effect on sustaining cognition over time (e.g., PreDIVA (17), MAPT (17)) and there is yet to be a clinical trial that demonstrates a reduction in dementia incidence as a result of individual-level multidomain lifestyle interventions (18).

In addition to these multidomain randomized controlled trials, public health initiatives aim to educate, raise awareness and provide the environmental opportunity and conditions for individuals to make lifestyle changes are also being rolled out (19, 20). For instance, a 10 month public health campaign in Netherlands, which utilized media and community participation, examined the campaign’s ability to raise awareness of modifiable dementia risk reduction and increase motivation for behavior change (19). Despite not reaching a population-level increase in awareness of dementia risk reduction, the study demonstrated that exposure to the campaign led to a greater awareness and motivation for behavior change. However, effective population-level prevention requires both individual motivation for behavior change, along with public health action supported by widespread societal and policy change which poses additional systemic challenges (6, 21, 22).

Despite the promise of developing and adopting public health campaigns to target behavior change for dementia risk reduction, these findings are from a substantially different context to the coronavirus disease 2019 (COVID-19) pandemic climate (23, 24). The COVID-19 pandemic and its associated restrictions and lockdowns have significantly affected the daily lives of all individuals, but particularly older adults (25–27). The multiple waves of COVID-19 restrictions on leisure and gatherings have reduced older adults’ rate of physical activity and social engagement (28), further exacerbating the severity of dementia risk factors (e.g., limiting physical activity, enforcing social isolation), and creating associated cascading effects on health and wellbeing (e.g., poor dietary choices, sleep problems, perceived stress) (29).

Only a few studies have explored how lifestyle behaviors may have altered during the pandemic. Bartlett and colleagues (30) demonstrated no detrimental effects of restrictions on the lifestyle behaviors of older adults before and during COVID-19 restrictions. However, these findings may be attributed to the 4-week dementia risk reduction program Preventing Dementia Massive Open Online Course (PD-MOOC) that the sample were engaged in at the time. Compared to the personalized, planning and action-oriented focus of BRAIN BOOTCAMP (31), the PD-MOOC is primarily educational, aiming to build knowledge of self-management of modifiable risk factors, thus our study will add to the evidence surrounding the protective nature of multidomain lifestyle programs.

Specific to brain health, Waterink and colleagues (32) found that 74% of participants reported at least one adverse lifestyle change, however, 60% of participants also reported at least one positive change including increased exercise, healthier food consumption and reduced alcohol consumption. These behaviors also differed based on sociodemographic factors such as age, gender, living circumstances, and income. Younger older adults, females, individuals living alone and in urban areas and individuals with an unsatisfactory income, reported more negative impacts on their lifestyle in a Dutch sample (32); whilst females, unemployed, retired and those reporting better adherence to restrictions, reported diminished physical activity in an Italian sample (33). Although long-term impacts of COVID-19 on lifestyle are unknown, research highlights the need to consider the sustained impacts of COVID-19 restrictions on vulnerable populations, including physical, financial and social recovery after COVID-19 restrictions and the fundamental changes in the way people think/behave and institutions operate. This is important within public health interventions or campaigns when designing and implementing methods to increase awareness, generate reductions in dementia risk, and encouraging older adults to remain brain healthy even in new and potentially adverse circumstances.

Understanding the impact of the COVID-19 pandemic on behavior change will be essential to informing the development of an effective dementia prevention intervention that targets the specific needs of older adults and is context-specific. Therefore, the aim of this qualitative study was to explore how the COVID-19 pandemic affected older adults’ habits and to provide strategies and recommendations for future developments that target dementia prevention in a post-pandemic era.

## 2. Methods

### 2.1. Participants

Participants were older adults residing in New South Wales (NSW), Australia, who had recently partaken in the BRAIN BOOTCAMP pre-post prospective intervention program for brain health, involving a lifestyle behavioral modification program to improve dementia literacy and reduce dementia risk among older adults. Further details on the program have been published elsewhere (31). Older adults were recruited using standardized advertising methods including flyer dissemination (e.g., in primary care clinics, memory clinics, local community newsletters, and radio) in NSW, Australia. The opportunity to participate was also provided to disadvantaged or vulnerable communities (e.g., low socioeconomic status, ethnic minorities) through larger organizations who keep a registry of pre-existing members willing to participate in such research. Older adults were eligible if they were 65 years of age or more and were community-dwelling. They were required to be English-speaking and without significant self-reported depressive episodes, existing diagnosis of dementia, inability or refusal to provide informed consent, or current registration in another lifestyle modification intervention.

### 2.2. Procedure

Originally, 853 participants were recruited for the intervention program between January 2021 and March 2021. All participants who completed the intervention program were invited to take part in a semi-structured telephone interview.

Telephone interviews were completed during the second COVID-19 restrictions in NSW, Australia, between 26 June to 11 October 2021 (15 weeks of COVID-19 restrictions in total). This was during the height of the pandemic in Australia and restrictions included lockdowns with participants unable to leave their homes except for essential work and exercise within 5 km of their local government area (34). Residents were not allowed to visit family or friends during this period and were only able to see those in their households. The majority of retail shops were closed except for grocery stores or retail outlets that had ‘click and collect’ available. Residents in NSW were unable to travel out of Greater Sydney and all state borders within Australia were closed. Schools were also closed during this time, with children requiring home-schooling. Community sport and other face-to-face recreational activities were put on hold for this period.

Researchers conducted semi-structured interviews as part of a broader mixed-method longitudinal study (31). Both researchers (JS, LD) were present for 85.7% of interviews. The purposive sampling considered the variability of participants in terms of age, current brain risk score (high and low modifiable dementia risk), gender (male and female), locality (major city or other) education (primary, secondary, tertiary), country of birth, primary language (English or other), and socioeconomic status (high and low). A semi-structured interview guide using a conversational style was developed by the research team and consisted of 4 key open-ended questions to guide the interview (see Supplementary material). The interviews began with a question regarding the impact of COVID-19 on the participant’s lifestyle behaviors, followed by questions surrounding their capability to adapt to the restrictions imposed by the pandemic, intended behavioral adaptations following the anticipated lifting of restrictions, and any suggested improvements or feedback future intervention programs for their ongoing brain health.

### 2.3. Qualitative data analysis

All interviews were audio recorded and transcribed verbatim for content analysis using combined assistive technology (e.g., Zoom, OtterAI). Transcripts were checked for accuracy by the moderator (JS) and research team (LD). Grounded theory (35) and the self-regulatory (SRM) framework (36) were used to evaluate the transcripts. A randomly selected 20% of transcripts were read independently by two researchers (JS, CB) and an initial framework was developed. Thematic data analysis was performed using an deductive approach by two researchers for the remainder of the transcripts (JS, CB). Although specific key research questions were answered and guided the analysis, open coding was applied with no pre-set codes; rather codes were developed from interpretation of the data and modified throughout the analysis as required. The general process of qualitative data extraction included familiarity with the data, initial coding of data, allowing key meaningful themes and sub-themes relevant to the study objectives to be extracted from the data. These were then reviewed, re-considered with respect to coding and study objectives and then adapted as necessary to form emergent themes. After internal discussions between JS and CB, the framework was further refined and applied to the remainder of the transcripts. NVivo V20 was used to manage the transcripts and assist with analysis.

## 3. Results

### 3.1. Characteristics of study sample

A total of 165 eligible urban-dwelling, older adults received an invitation to participate in the telephone interviews for the present study, of which 159 (96.4%) accepted the invitation and completed a semi-structured telephone interview. Demographics of the included study participants are listed in Table 1.

**Table 1:** Sociodemographic characteristics of participants (*N* = 159).

Characteristic	*N* (%)
**Gender**	
Female	118 (74.7)
Male	40 (25.3)
**Age (mean [SD], range)**	73.1 [5.6], 65–90
65–69	50 (31.7)
70–79	87 (55.1)
80+	21 (13.3)
**Country of birth**	
English-speaking country	141 (89.2)
Non-English speaking country	17 (10.8)
**Education in years (mean, [SD], range)**	9.5 [1.7], 0–30
**Socioeconomic status (quintile)**	
1 (lowest)	12 (7.6)
2	21 (13.4)
3	24 (15.3)
4	14 (8.9)
5 (highest)	86 (54.8)
**Locality**	
Metropolitan	129 (82.2)
Regional	28 (17.8)
Modifiable dementia risk (LIBRA index) (mean [SD], range)	−3.1 [2.5], −5.9–3.1

LIBRA, LIfestyle for BRAin Health index; SD, standard deviation.

### 3.2. Themes

**Figure 1:**
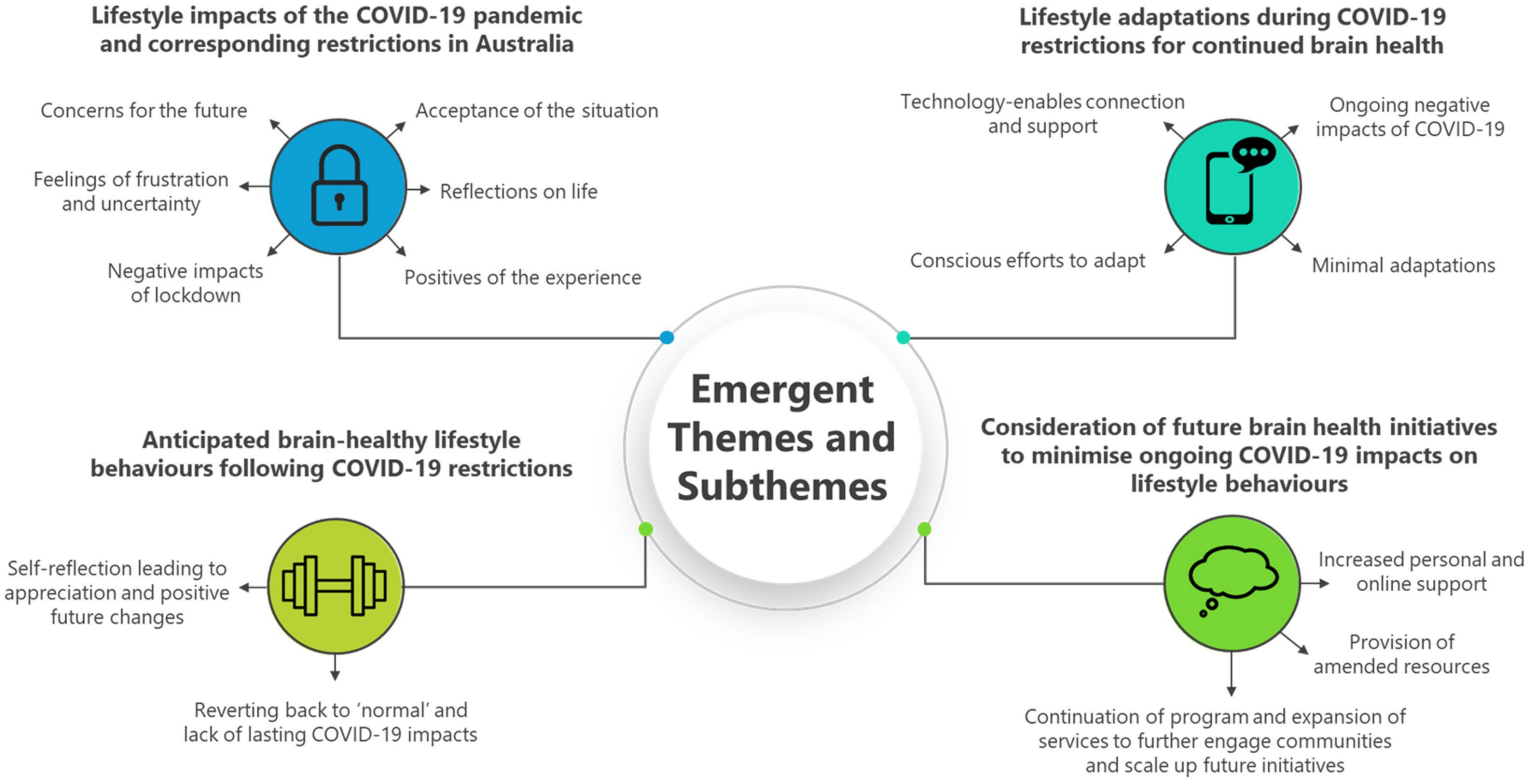
Thematic framework of emerging themes and sub-themes following analysis.

Through deductive thematic coding and data analysis, emergent themes were grouped into four main themes and fifteen subthemes. The four main themes were: (1) Lifestyle impacts of the COVID-19 pandemic and corresponding restrictions in Australia; (2) Lifestyle adaptations during COVID-19 restrictions for continued brain health; (3) Anticipated brain-healthy lifestyle behaviors following COVID-19 restrictions; and (4) Consideration of future brain health initiatives to minimize ongoing COVID-19 impacts on lifestyle behaviors. Each theme is discussed in more detail below, with specific examples from each sub-theme and corresponding direct quotes from study participants as evidence of the deductive approach adopted for analysis. Furthermore, a diagrammatic representation of emerging themes and sub-themes is visually displayed as a theoretical framework in Figure 1.

#### 3.2.1. Theme 1: lifestyle impacts of the COVID-19 pandemic and corresponding restrictions in Australia

Participants provided a range of descriptors relating to impacts of the COVID-19 pandemic and its associated restrictions on their lifestyle. Such descriptors included concerns for the future, feelings of frustration and uncertainty, negative impacts of lockdown, positives of the experience, reflections on life and acceptance of the situation. Some of these were positive or neutral, while others were entirely negative, or a combination of both positive and negative. Participants who described concerns for the future primarily discussed feelings of worry, skepticism and sadness. They expressed worries and fears about what the future would look like for the next generations and considered if the present situation would end up becoming permanent as a ‘new normal’.

*“I would wake up and feel really sad and I would say what kind of a world are my grandchildren coming into? And this is my worry for the future of my children and my grandchildren.” (P24).*

The restrictions imposed by the COVID-19 pandemic also created feelings of frustration and uncertainty for many participants. Many expressed becoming increasingly lazy as a result, which for some was an unexpected feeling, and was possibly brought on from a lack of motivation.

*“And I find I sit around a lot more which is not quite so good. Although I am a competitive sports person, my default position is laziness. And lockdown invites laziness.” (P30).*

Many respondents reported negative impacts on lifestyle factors that support brain health. Some felt that they had less motivation to exercise from home and stated that the environmental impact of not being able to access exercise facilities reduced the ability to stay physically healthy (e.g., gyms, fitness classes).

*“It has obviously made it impossible to continue the activities I felt were keeping me physically healthy, like Pilates and going to the gym.” (P415).*

Others noted that they had put off necessary health tests, or developed bad habits around their diet, such as an increase in alcohol consumption.

*“I was due for blood tests and a bone density scans, just physical health issues. And I have been putting them off and putting them off because I did not want to go into that environment.” (P470).*

*“We had started to drink more.” (P114).*

Furthermore, environmental restrictions substantively influenced social lifestyles, with respondents missing the social aspect more than any other element, describing it as a critical part of their lives. Although most were able to maintain regular contact with family and friends through the telephone and other teleconferencing platforms such as Zoom, some individuals were not able to socialise and described it as a huge loss. Others also expressed their dislike of the reliance on technologies such as Zoom as it did not replace real life face-to-face interaction:

*“A lot of things have gone on Zoom, and I find Zoom very unsatisfactory as it feels like you are not talking to real people, like you are looking at an album or postage stamps.” (P615).*

However, not all participants felt the COVID-19 restrictions were entirely negative. Many described positive impacts of the experience, such as how the allowance of individual time due to restrictions permitted a conscious effort to increase their mental stimulation and opportunities for familial socialization. They described feelings of togetherness and gratitude for the individual situation they were in. Some found the imposed lockdowns useful since COVID-19 could be a good excuse to cancel social plans. Others also used this additional time at home to achieve personal goals and develop new skills.

*“I mean it’s changed my lifestyle but not in a bad way. I’m probably walking more in COVID and sometimes I walk for an hour and a half and do 2000 steps which I did not have time to do before COVID.” (P583).*

#### 3.2.2. Theme 2: lifestyle adaptations during COVID-19 restrictions for continued brain health

Participants discussed a variety of adaptations to adapt to the COVID-19 situation in order to continue to care for their brain health. Many described conscious efforts to adjust to the situation, such as through new use of technologies to enable ongoing social connection and personal support.

*“I just wanna be as healthy and fit as I can be in any circumstances.” (P224).*

A large proportion of participants described efforts to adapt around increasing or continuing exercise, particularly around walking, which for some has also meant they can have some social connection at the same time.

*“You can only meet up with one person at a time, and fortunately I have a friend I walk with every day.” (P456).*

Other forms of mental stimulation frequently described by participants included word puzzles and Sudoku, reading, writing, planning and ongoing study. Others have also been exposed to home schooling or trying new or more challenging hobbies.

*“I love cryptic crosswords. I do cricket crosswords. I do code breakers. I do a Master of Research.” (P35).*

In response to COVID-19 restrictions, respondents stated that they primarily adapted their lifestyle by utilizing more technology and practicing conscious motivational efforts when it came to maintaining or increasing healthy brain activity. The majority of respondents noted that through the use of technology, they were able to keep some social connections going through restrictions with family and friends via Skype, Zoom, emailing and more.

*“I’m socially active with friends and different groups, either by email or Facebook or phone calls.” (P40).*

Others stated that the restrictions had influenced them to use technology to support everyday activities such as online shopping.

*“For the first time ever, buying food online rather than go to the local store.” (P9).*

Conscious motivational efforts were a primary adaptive response to the COVID-19 restrictions, particularly with mental stimulation which ranged from puzzles, crosswords, painting, knitting, reading and online games.

*“I am doing a bit more in the sphere of crosswords and games, and online problem-solving as well.” (P415).*

However, many participants continued to describe the negative impacts of COVID-19 and associated restrictions on their ability to adapt. Some of these reasons were ongoing reductions in motivation to exercise, not wanting to go outside of their comfort zone to try new things, regressing behaviors and simply not adapting well to the drastic changes imposed upon them. Others made little to no conscious effort to adapt their lifestyle behaviors to maintain optimal brain health.

*“I’ve been exercising a bit less. The classes in the gyms and things have closed down. It’s mainly just walking now, and I do not really have the motivation to exercise at home, it’s not the same …. The first time, yes, it was all zoom and house party, but the novelty has worn off this time.” (P44).*

*“I’m not using my brain at the moment darl, it’s made me lazy you know.” (P806).*

#### 3.2.3. Theme 3: anticipated brain-healthy lifestyle behaviors following COVID-19 restrictions

Participants described that COVID-19 had resulted in moments of self-reflection. Such contemplative moments had created feelings of appreciation and the determination to make positive future changes to their lifestyles once restrictions had eased. A common thread among participants would that they would do more, such as increasing travel and social activities, be outside more, do more physical activity and eat out more often. They also reflected on feelings of gratitude that life had not always been like this.

*“It’ll be just wonderful to just to do all those normal things that we took for granted.” (P470).*

While many expressed the desire to just be able to do more again, some participants described that they might do things a bit differently, such as exercising caution and hesitation in what may become a new ‘normal’. This was particularly apparent around continuations of COVID-safe measures and the acknowledgement that an ease of restrictions may not mean life returns to normal for them.

*“I think I’ll probably always wear a mask on public transport for the rest of my life. I feel naked without one.” (P470).*

There was a sense that life would simply revert back to normal and previous activities would be able to be resumed. This was a conscious decision at times to start returning to activities participants were doing before the COVID-19 lockdowns, but among others they were simply waiting until restrictions were eased and programs were started up again. A common theme in the response to continual lifestyle adaptations post COVID-19 restrictions primarily fell under a reversion back to normal, pre-COVID-19 lifestyles, or adaptation to future changes as they arrive.

*“I’ll certainly go back to doing all the things I did before, I hope.” (P775).*

*“After lockdown I’ll keep the brain going … I bought myself one of those electronic chess sets so that certainly stimulates my brain … I would get back to playing table tennis which I normally do 3 days a week so I’ll get back into that.” (P114).*

*“I’ve always been active. I’ll continue that when we get back to the real life again.” (P425).*

The potential lasting impacts of COVID-19 could not be ignored by some however, as they expressed opinions that things would not change very much even after restrictions eased.

*“Probably it will not change a great deal, I have a son who lives in England and I’m not getting over there soon, so restrictions would probably have to ease a lot before it made a big change to me.” (P464).*

#### 3.2.4. Theme 4: consideration of future brain health initiatives to minimize ongoing COVID-19 impacts on lifestyle behaviors

In response to potential ongoing negative impacts of COVID-19 on brain-healthy lifestyle behaviors, participants provided suggestions to increase the personal and online support of future lifestyle programs.

*“Maybe there could be a particular section on how you can keep in contact with people for example Zoom, Facetime and things, maybe just have it set down and some links for specific help that you could apply for if you were impacted from COVID-19.” (P308).*

Most respondents stated that adding social contact would bring benefits as it will enable substantial support of the impacted health and lifestyle behaviors as a consequence of COVID-19.

*“I think the checking in on people is good. You know, “Hi, this is [the research program]. How are you going?” It kind of makes us go, oh right, yes, I better do something about that.” (P70).*

Some respondents highlighted the value of regular phone calls or setting up of support groups to motivate and improve the social aspect of brain health. There were suggestions of greater online resources, more advice and more check-in times during the program, or the implementation of a buddy system which can help support individuals to be more socially involved.

*“Buddy up with someone else who’s doing it and grab a coffee with them.” (P464).*

Others described the need to have stronger connections with local organizations and community groups to foster engagement and support.

*“I think you could have more connection with local organisations, like council.” (P24).*

Some respondents emphasized the importance of individual agency and choice in life. They believed that people have the power to individually shape their own lives and achieve their goals if they have the desire to do so.

*“I think it’s up to the individual, but if you want to do things, you can. It’s a matter of getting people to take an interest in those things and try and keep themselves healthy as long as you can.” (P529).*

Most respondents reflected a more uncertain and open-minded perspective. Some individuals were unclear about future directions and suggested that the topic might be better positioned within the research team as well as the diversity of individual needs and preferences, respecting individual differences and indicating reluctance to impose a one-size-fits-all approach.

*“Oh, I really do not have an idea there really. I do not know, I honestly do not know. It depends on the individual, does it not? Some people would benefit from a bit more contact, others not, myself personally, I’m fine.” (P542).*

## 4. Discussion

### 4.1. Main findings

The COVID-19 pandemic has affected the lifestyles of community-dwelling older adults in Australia with enduring influence on lifestyle restrictions which can impede brain health. The nature of identified impacts varied widely and included positive influencers (increased time at home for exercise, cooking and hobbies), however participants also expressed overarching negative impressions on their lifestyles, which centered on social isolation from stay-at-home orders, and feelings of frustration and uncertainty.

### 4.2. Findings in the context of existing evidence

#### 4.2.1. COVID-19 and brain healthy lifestyles

Previous international research has identified certain aspects of our lifestyle that have been impacted by the pandemic. A worldwide online survey disseminated across Europe, North Africa, Western Asia and the Americas, found social participation and physical activity had reduced by 42 and 24%, respectively, from pre-confinement to during confinement periods (37). Sleep quality was also reduced during this time, healthy eating habits decreased as well as a number of other measures of wellbeing (i.e., emotional status, life satisfaction) (37). Respondents also identified an unhealthy reliance on technology. There are similarities in our findings with these results and the qualitative nature of our study may offer avenues of supporting brain health, as well as general health through targeted brain health interventions, given that many areas of health are modifiable risk factors for dementia.

COVID-19 restrictions impacted negatively on the social lifestyles of older adults. This is a common finding among other studies (26, 27, 38) and is of particular concern as social isolation may contribute to exacerbation of dementia risk factors, including mental health whilst limiting social opportunities found in physical activities. It is known that an active lifestyle incorporating both physical and social activity is necessary for optimal brain health (28). There is also potential to reduce dementia risk by enhancing or maintaining cognitive reserve, and frequent social contact is a named factor (3). Given the protective nature of social connection and the importance of social integration within lifestyle modifying programs, future intervention studies to reduce modifiable dementia risk factors will need to consider uncertainties associated with emerging from social isolation alongside hesitation to participate in large social events and activities due to apprehension of contracting COVID-19.

Another detrimental impact consistent with previous research is reduced physical activity levels during home confinement (28, 37). The reductions on all levels of physical activity and greater amounts of time sitting daily (39) is described in several cross-sectional studies in Dutch, Japanese, Italian and Australian older adult samples (32, 33, 40, 41). Our study expands on this and provides insights into the personal challenges involved in adapting and managing their lifestyles to foster physical activity under these new circumstances. Participants who expressed a conscious effort to adopt strategies to negate unhealthy behaviors often focused on physical and cognitive activity, suggesting that certain brain healthy behaviors can be easily substituted and maintained compared to others. Identifying and supporting commonly accepted risk factors will have important implications for future single and multidomain dementia risk reduction programs post pandemic.

#### 4.2.2. Ongoing impacts and adaptations after COVID-19

There were concerns expressed on the enduring effects that COVID-19 may have in the future. Our results found that during the restriction period, a large proportion of older adults made changes to adapt to the situation, such as through the use of technology and online resources, as well as ensuring interaction and mental stimulation from games, puzzles and academic pursuits. Most made deliberate attempts to adapt their exercise regime too, such as increasing walking or partaking in online exercise classes. While some participants had adjusted their lifestyle to continue to be brain healthy, others showed little adaptation, having made almost no changes to their lifestyle, and found the impacts of COVID-19 and associated restrictions overwhelming and struggled to maintain healthy habits.

Broader COVID-19 research suggests that this could be a direct health consequence of acquiring COVID-19 with pre-existing chronic conditions (respiratory, cardiovascular, neurodegenerative diseases) which creates persistent symptoms, increased frailty, poorer health and can cause long COVID-19 (42) amongst older adults. Reductions in brain healthy lifestyle may also be a characteristic of those who exhibited higher adherence to government orders (33), those with pre-existing mental health conditions (anxiety, depression and post-traumatic stress disorder) (43), subjective memory complaints (32), and those who reported difficulties with certain behaviors like sleep problems and elevated alcohol and drug use (43) throughout lockdown which may have had a compounding effect on their ability to stay engaged in a healthy lifestyle.

Following COVID-19 restrictions, for the minority of participants there was a clear eagerness to socialize and travel as soon as restrictions eased, while others exhibited feelings of caution and hesitation, which is a common finding alongside fear, avoidance and procrastination (38, 43, 44). Our results depict insights into older adults’ perspectives on their lifestyles in the hope of restrictions easing and whether that would reveal a ‘new normal’ or if life would return to pre-COVID conditions. There were a range of expected approaches to removal of restrictions shown among the participants. This included actioning their sense of acknowledgement and renewed appreciation of life by expressing motivation to sustain the new habits that had put in place, whilst others assumed they would just resume previous activities or remained largely unaffected by the situation due to their place of residence (e.g., rural areas with minimal restrictions).

Post COVID-19 research suggests that these behaviors during confinement are critical in remaining consistent with healthy behaviors after lockdown. For example, in one study older adults who were physically active during lockdown are more likely to facilitate exercise post lockdown (45). In another, the learning of new technologies during the stay at home period increased intention to use social networking sites, with a jump from 27% pre-restrictions to 50% after restrictions eased (46). Both findings suggest significant potential to propel healthier outcomes associated with exercise and connectivity in the post lockdown context (47).

### 4.3. Implications

Suggestions for future brain health (intervention) programs included greater and varied online resources with content and advice around healthy lifestyle choices. Program design and structural feedback included more personal support by way of an e-newsletter or more phone calls for encouragement. A final suggestion was to expand and scale-up future programs to reach more diverse groups of people and using the program as a way to create more intimate and engaged communities.

### 4.4. Limitations

Our study had various limitations including the nature of recruitment and the telephone-based format. Many older adults were already interested in completing a brain health intervention program, participation in the interview was optional and the restrictions did not allow for face-to-face interviews which provide non-verbal and contextual data that could have contributed deeper meaning to participant responses (48). Although we recruited through varied mediums including print (flyers), radio and television this may have led to a selection bias (highly educated, high socioeconomic status, lower modifiable dementia risk) and this means that our findings are not generalisable to the most vulnerable older adult populations with less (brain) healthy lifestyles and higher dementia risk. The strengths of our study are the timing of our data collection, the large sample size and high response rate (96.4%). This enabled us to explore older adults insights during the second and perhaps harsher COVID-19 lockdown period in Australia (26), rather than relying on participant recall of events. It also allowed for a variety of responses in which common themes were identified despite the variations in locality and experiences.

## 5. Conclusion

Considering our findings and the early evidence for the effectiveness of multidomain interventions targeting changes in lifestyle behaviors in delaying cognitive decline (4, 5, 15–18, 49), development and implementation of dementia risk programs should consider the short and long-term impediments and opportunities for lifestyle change amongst individuals, communities and healthcare systems in the post COVID-19 context. Given the reported challenges involved in maintaining a brain healthy lifestyle throughout the pandemic and likely ongoing ramifications, future action should seek to involve policy change to support and bolster the potential impact of multidomain interventions, especially as older adults adapt to the new ‘normal’.

## Data availability statement

The original contributions presented in the study are included in the article/Supplementary material, further inquiries can be directed to the corresponding author.

## Ethics statement

The studies involving humans were approved by Macquarie University Human Ethics Committee. The studies were conducted in accordance with the local legislation and institutional requirements. The participants provided their written informed consent to participate in this study.

## Author contributions

JS: conceptualization and funding acquisition, writing-original draft preparation, and supervision. JS and CB: methodology and formal analysis. JS, LD, and KD: validation. JS and LD: investigation and project administration. JS, LD, CB, and KD: writing-review and editing. All authors contributed to the article and approved the submitted version.

## Funding

This article presents independent research funded by the New South Wales Government, My Community Project Grant number MCP19-04026 to JS.

## Conflict of interest

The authors declare that the research was conducted in the absence of any commercial or financial relationships that could be construed as a potential conflict of interest.

## Publisher’s note

All claims expressed in this article are solely those of the authors and do not necessarily represent those of their affiliated organizations, or those of the publisher, the editors and the reviewers. Any product that may be evaluated in this article, or claim that may be made by its manufacturer, is not guaranteed or endorsed by the publisher.
