# Supplementary material for: Older adults’ perspectives towards optimizing lifestyle behaviors and strategies to support healthy brain ageing during COVID-19 restrictions
Source: Front Public Health. 2023 Aug 30;11:1205001. doi: 10.3389/fpubh.2023.1205001 (PMC10499331; doi:10.3389/fpubh.2023.1205001)
Supplement: Supplementary file 1 [file Table_1.DOCX]

Supplementary Material

“Lockdown invites laziness”: Older adults’ perspectives towards optimizing lifestyle behaviors and strategies to support healthy brain ageing during COVID-19 restrictions

Joyce Siette^1,2*^, Laura Dodds^1^, Cristy Brooks^3^, Kay Deckers^4^

*** Correspondence:** Dr Joyce Siette: [joyce.siette@westernsydney.edu.au](mailto:joyce.siette@westernsydney.edu.au)

# Interview Guide

• Do you feel that COVID-19 had an impact on your health and lifestyle behaviors? Was it positive or negative? What was your lifestyle like before COVID-19?

• During COVID-19 restrictions (e.g. lockdowns this week/last week) how have you adapted your lifestyle to continue to be brain healthy? Have you experienced changes with healthcare checks such as visits to General Practitioner, being physically active [how they have managed this during lockdowns], being socially active [how], and other risk factors?

• Will you adapt your lifestyle following COVID-19 restrictions? How so?

• Do you feel that existing dementia risk initiatives can help you sustain and commit to your goals for a healthier lifestyle? Why or why not?

• How could future dementia risk reduction initiatives be improved to minimize the negative impact of COVID-19 on health and lifestyle behaviors?

• What are your thoughts on additional resources (what type) and more involvement from different people (family, local organizations)?
